# Disk injury in patients with vertebral fractures—a prospective diagnostic accuracy study using dual-energy computed tomography

**DOI:** 10.1007/s00330-018-5963-4

**Published:** 2019-01-16

**Authors:** Matthias Pumberger, Michael Fuchs, Nils Engelhard, Kay Geert Hermann, Michael Putzier, Marcus R. Makowski, Bernd Hamm, Torsten Diekhoff

**Affiliations:** 1 Department of Spine Surgery, Center for Musculoskeletal Surgery, Charité - Universitätsmedizin Berlin, Campus Mitte, Humboldt-Universität zu Berlin, Freie Universität Berlin, Berlin, Germany; 2 Department of Orthopedic Surgery, University of Ulm, Ulm, Germany; 3 Department of Radiology, Charité - Universitätsmedizin Berlin, Campus Mitte, Humboldt-Universität zu Berlin, Freie Universität Berlin, Charitéplatz 1, 10117 Berlin, Germany

**Keywords:** Fractures, compression, Tomography, X-ray computed, Spine, Collagen

## Abstract

**Objectives:**

Using magnetic resonance imaging (MRI) as the standard of reference, we aimed to evaluate the diagnostic accuracy of dual-energy computed tomography (DECT) in assessing disk injuries in patients aged more than 50 years with vertebral fractures.

**Methods:**

This prospective study was approved by the local ethics committee (EA1/372/14), and all patients gave written informed consent. Patients with suspected fractures underwent spinal DECTs and MRIs. Three readers scored DECT collagen maps for the presence or absence of disk injuries and also scored MR images according to the Sander classification (0–3). Only disks at risk (target disks) were included in the analysis. Sensitivity and specificity were calculated. Fleiss’s *κ* was used to evaluate interrater agreement. Attenuation, in Hounsfield units, was compared between affected and unaffected disks in DECT.

**Results:**

Analyzing 295 disks in 67 patients, DECT was both sensitive (0.85) and specific (0.75). Sensitivity varied with the severity of disk damage, as assessed using the Sander scale (grade 1, 0.80; 2, 0.85; and 3, 0.98). Fleiss’s *κ* was 0.41 for MRI and 0.51 for DECT. In the DECT collagen maps, attenuation was lower in injured disks compared to that in normal disks (80.3 ± 35.2 vs. 97.9 ± 41.0, *p <* 0.001).

**Conclusions:**

Compared to conventional CT, DECT collagen maps can yield more diagnostic information, allowing identification of disk injuries in elderly patients with vertebral fractures.

**Key Points:**

*• Dual-energy computed tomography allows vertebral disk injuries to be detected in elderly patients with vertebral fractures.*

*• Dual-energy computed tomography yields more diagnostic information about vertebral disks compared to conventional CT.*

• *Dual-energy computed tomography can be used as an alternative imaging modality for patients unwilling or unable to undergo MRI.*

**Electronic supplementary material:**

The online version of this article (10.1007/s00330-018-5963-4) contains supplementary material, which is available to authorized users.

## Introduction

Treatment of thoracolumbar spinal fractures, whether conservative or surgical, remains controversial. In 2005, a novel classification system, the Thoracolumbar Injury Classification System, was established, taking into account not only the fracture mechanism and posterior ligamentous complex injuries, but also the patient’s neurological status [1]. Several years later, AOSpine combined it with the older Magerl classification system, forming the AOSpine Thoracolumbar Spine Injury Classification System [2, 3].

These classification systems are gaining popularity worldwide, and they assist surgeons in deciding when surgery is indicated and if it is the best approach. However, the critical aspects of injuries to the intervertebral disks have not yet been addressed. Disk injuries can be detected in magnetic resonance imaging (MRI) as high signal intensities in T1- or T2-weighted sequences [4] with loss of the nuclear cleft. Particularly when fracture patterns indicate potential disk injury, preoperative imaging could be a decisive factor. Given that patients with traumatic spinal injuries are relatively young, long-term survival and restored function of the vertebral disk are crucial [5]. Traumatic disk injury leads to decreased nutrient supply, the primary cause of disk degeneration [6, 7]. Whereas conservative treatment or transpedicular spinal fusion could suffice in exclusive osseous lesions, accompanying disk injuries often trigger the need for discectomy or corrective spinal fusion. Interestingly, combined injury to the superior endplate and vertebral disk is associated with a greater complication rate even in patients undergoing surgery [8]. Although some surgeons already take this fact into account, timely availability of cross-sectional imaging, MRI in particular, remains limited in many hospitals. However, compared to MRI, CT is supposed to be cheaper, faster, and less discomfortable and has fewer contraindications.

Dual-energy computed tomography (DECT) is an established imaging technique first introduced in the fields of rheumatology and urology [9, 10]. Special postprocessing tools allow detection of bone marrow edema using the so-called virtual non-calcium technique [11]. When a three-material decomposition algorithm is applied, DECT also allows visualization of collagenous structures such as tendons and ligaments [12, 13]. In this study, we applied this technique to the vertebral disk. All patients also underwent MRI, which served as a reference standard to confirm or rule out acute disk injury.

Because computed tomography (CT) remains the diagnostic gold standard for detecting fracture morphology, the aim of this prospective diagnostic accuracy study was to evaluate the diagnostic value of DECT for identifying vertebral disk injuries in patients with spinal fractures evidenced in CT scans.

## Methods

### Patients

Between January 2015 and February 2017, we prospectively and consecutively enrolled patients aged more than 50 years presenting with acute back pain and vertebral fractures visible on radiographs. Exclusion criteria were contraindications to MRI and the inability to give informed consent. Patients included in this study were also used in an evaluation of bone marrow edema, reported elsewhere [14].

### Imaging

All patients underwent DECT and MRI of the thoracic or lumbar regions of the spine. The former was performed on a 320-row single-source machine (Canon Aquilion ONE Vision Edition; Canon Medical Systems), and it included both a scanogram and a sequential volume acquisition of two energy datasets (135 and 80 kVp). Rotation time was 0.275 s and the change-over time was 0.5 s. A standard deviation (SD) value of 12 Hounsfield units (HU) was applied as a noise equivalent for automatic exposure control at both energy levels, resulting in a mean (SD) dose length product of 582.3 (163.9); the computed tomography dose index volume was 16.2 (1.0). The datasets were reconstructed using a slice thickness of 0.5 mm and a medium soft tissue kernel without beam hardening compensation (FC13) but with the iterative reconstruction (AIDR-3D) standard. Virtual collagen maps were reconstructed in 3-mm sagittal slices on the CT console (Dual Energy Image View, Version 6; Canon Medical Systems) using a collagen-specific gradient of 1.10. Two kinds of collagen maps were reconstructed: a black-and-white reconstruction for density measurement and a 50% color-coded fusion image for image reading, which provides better anatomical correlation. Additionally, morphological sagittal CT images were reconstructed from the 135-kVp dataset at a slice thickness of 3 mm using a bone reconstruction kernel.

Each MRI was performed using a clinical 1.5-T standard imager (MAGNETOM Avanto; Siemens Healthineers or MAGNETOM Symphony Vision; Siemens Healthineers) and included both the T1-weighted (repetition time, 551 ms; echo time, 12 ms; scan time, 5 min 12 s) sequence and a short tau inversion recovery (STIR) sequence (repetition time, 6150 ms; echo time, 31 ms; inversion time, 150 ms; scan time, 4 min 15 s) at a slice thickness of 3 mm.

Both images were examined as soon as they became available. Therefore, most patients underwent DECT first (e.g., directly and while at the emergency department) and MRI during the following days. When available first, MRI was performed before DECT.

### Target and reference disks

To obtain clinically relevant data without artificially increasing sample size, we did not include all disks, but only those at risk for damage. Disks in vertebral units (each consisting of a disk and half of the two vertebrae adjacent to it) that included a fractured vertebra were defined as target disks. A disk was included in the analysis only if it and the adjacent vertebrae were depicted in both imaging modalities. Fractured vertebrae were defined in a separate consensus reading by all readers prior to image reading using the bone-kernel-reconstructed CT datasets. Thus, fractures were included irrespective of fracture age. For this task, we transferred the Genant score [15] from radiography to CT to increase diagnostic accuracy.

To measure HU values in DECT sequences and signal intensities in MRI sequences, reader 3 defined a reference disk for each target disk which was the most caudal disk depicted by both MRI and DECT and appearing normal in both MRI sequences. Reference disks were selected separately for the thoracic and lumbar regions. The disk between the last thoracic and first lumbar vertebra was counted as a lumbar disk for this analysis. If no reference disk could be assigned as the target disk (e.g., because all disks were defined as target disks), the target disk was excluded from objective image analysis.

### Image reading

Three readers (reader 1, a radiologist specializing in musculoskeletal diseases with 8 years of experience; reader 2, a trauma surgeon with 8 years of experience; and reader 3, a research student with 1 year of experience) independently analyzed the separately anonymized MRI and DECT images. Disk changes on MRI were scored using the validated 4-point semiquantitative grading system according to Sander et al: 0, normal; 1, abnormally high signal in STIR; 2, high signal in T1; and 3, herniation [4]. A grade 1 was characterized by loss of the intranuclear cleft in T2-weighted images [16]. In DECT, disks were dichotomized, differentiating normal disks with high HU values in collagen maps (0) versus affected disks with low HU values (1), taking into account the air inside the disk or herniation. While the readers evaluated all disks depicted by the modality and were not aware of which disks were classified as target disks, only target disks were included in the analysis.

Additionally, the readers evaluated fracture morphology for endplate involvement of the fracture line in a dichotomized manner (0, no endplate involvement; 1, endplate involvement). This analysis was performed separately for cover and base plates.

The readers had access to all images of a given imaging modality during scoring, but were blinded to all clinical data and other imaging findings.

### Objective image quality parameters

Reader 3 measured target and reference disks for MRI-T1, MRI-STIR, conventional CT, and DECT collagen maps using a polygonal region of interest (ROI). The ROI included as much of the disk as possible in a central sagittal slice while maintaining a distance of at least 1 mm to the cortical bone and surrounding soft tissue. Mean signal intensities and attenuation values for MRI and DECT, respectively, were recorded.

Anonymization, scoring, and measurements were performed on a workstation with a high-resolution monitor using OsiriX (Version 6.4; Pixmeo SARL).

### Data analysis

A vertebral disk in MRI was classified as positive when a score of 1 or greater was assigned. For the analysis of all three readers combined, a finding in MRI or DECT was considered positive when two of the three readers agreed it was positive. A Wilcoxon matched-pairs signed-rank test was used to compare disk involvement and endplate involvement as depicted by MRI and DECT. Sensitivity (SE), specificity (SP), positive predictive value, and negative predictive value were calculated separately for each reader and all three readers taken together using contingency table analysis and MRI as the standard of reference. Furthermore, in DECT, the sensitivities of various grades according to Sander et al (rounded mean of the readers) were calculated. A subgroup analysis was conducted including patients under the age of 65 only.

Another contingency analysis was performed for endplate involvement using CT as the standard of reference. A phi correlation matrix was applied to compare disk positivity and endplate involvement between the modalities. Differences in attenuation, HU, and signal intensities of target disks and their corresponding reference disks in CT, DECT, MRI-T1, and STIR were calculated as measures of difference from normal tissue. These numbers were compared for both groups (affected disks vs. unaffected disks on MRI) using an unpaired *t* test for all disks and lumbar and thoracic disks separately. Interrater agreement in each modality was tested using Fleiss’s *κ*. Missing values (e.g., when no reference disk could be defined) were excluded from the analysis. The statistical analysis was performed using GraphPad Prism Version 7.0d and GraphPad Quick Calcs (GraphPad Software).

### Ethics approval and consent

This study was approved by the local ethics committee (EA1/372/14), and all patients gave written informed consent. Authorization by the German Federal Office of Radiation Protection (BfS) was waived by the ethics committee and the German Radiological Society (DRG).

## Results

### Patients

**Fig. 1:**
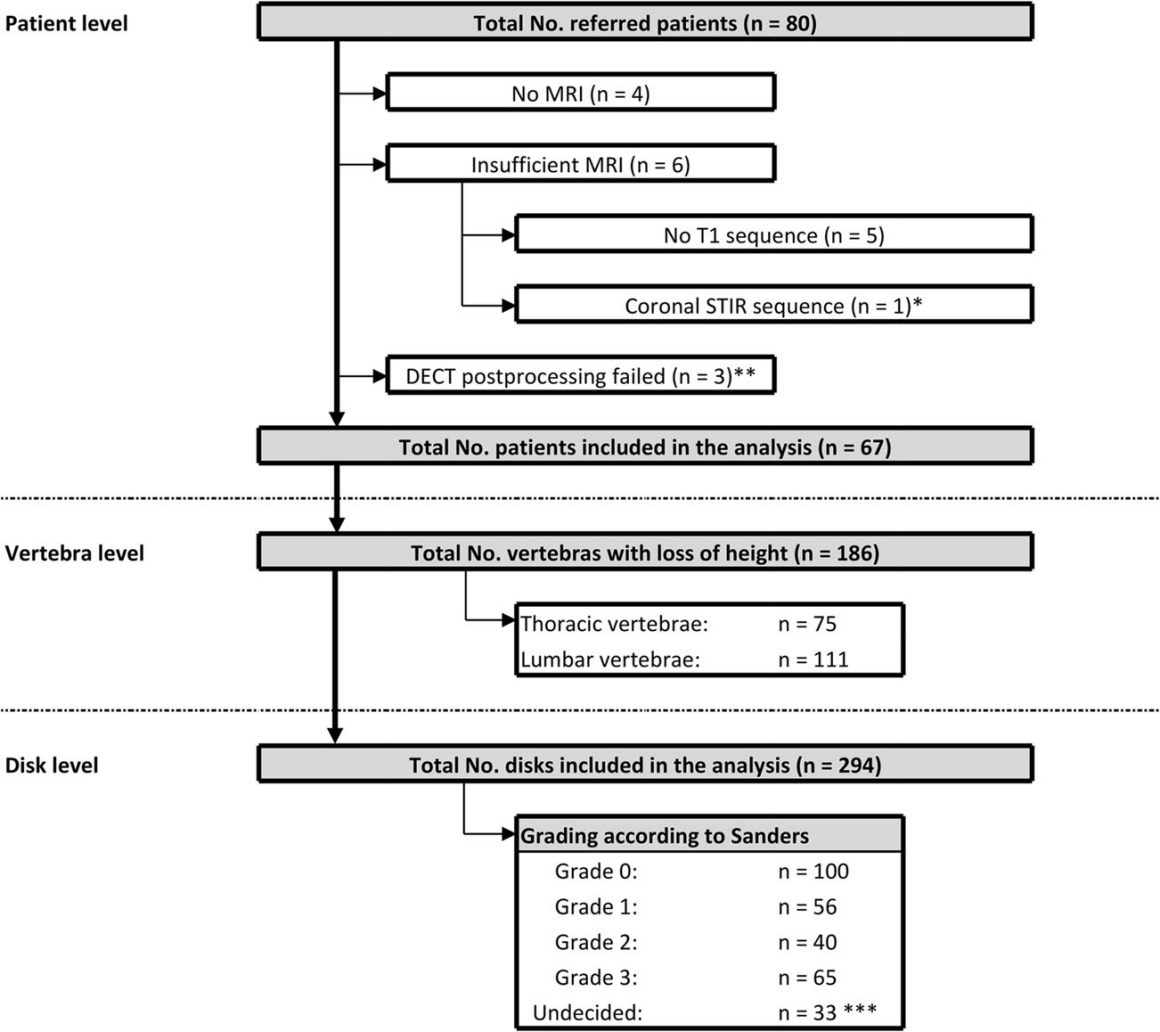
Patient flowchart. A total of 80 patients were imaged using dual-energy computed tomography (DECT), and 13 were excluded because of lacking or incomplete magnetic resonance imaging (MRI) data or failure in DECT postprocessing. The remaining 67 patients were included in the analysis, providing a total of 192 vertebrae with loss of height identified. A total of 294 adjacent disks were defined as target disks and included in the analysis. *One patient was excluded because of incorrect slice orientation of the short tau inversion recovery (STIR) sequence (coronal instead of sagittal). **Three DECT datasets were not accepted by the postprocessing software. ***Lacked agreement between at least two readers

Of the 80 patients who underwent DECT, 67 could be included in the analysis. Mean (SD) age was 70.7 (9.8) years. The median interval between DECT and MRI was 2 days; the mean was 4.4 (9.0) days. Both examinations were tolerated well by all patients. A flowchart of patient inclusion is shown in Fig. 1.

### Target disks and endplate involvement

A total of 295 disks were included in the analysis, 190 in the lumbar region and 105 in the thoracic region. Scoring positive in MRI was 194 disks, but in DECT, it was 192 disks (*p* = 0.59). At least two of the three readers assigned a score of 1 (high STIR signal) to 56 disks, of 2 (high T1 signal) to 40 disks, and of 3 (herniation) to 65 disks. Readers did not agree on the scores for 33 disks (see Fig. 1).

Using MRI, endplate involvement was detected in 142 vertebral units, but it was detected in 174 units using CT (*p* < 0.001). Endplate involvement found using CT correlated better with disk damage in MRI than endplate involvement found using MRI (phi coefficient, 0.49 vs. 0.41). However, disk injury in DECT correlated best with endplate involvement in CT (phi coefficient, 0.51 for CT vs. 0.36 for MRI endplate involvement).

### Contingency analysis

**Fig. 2:**
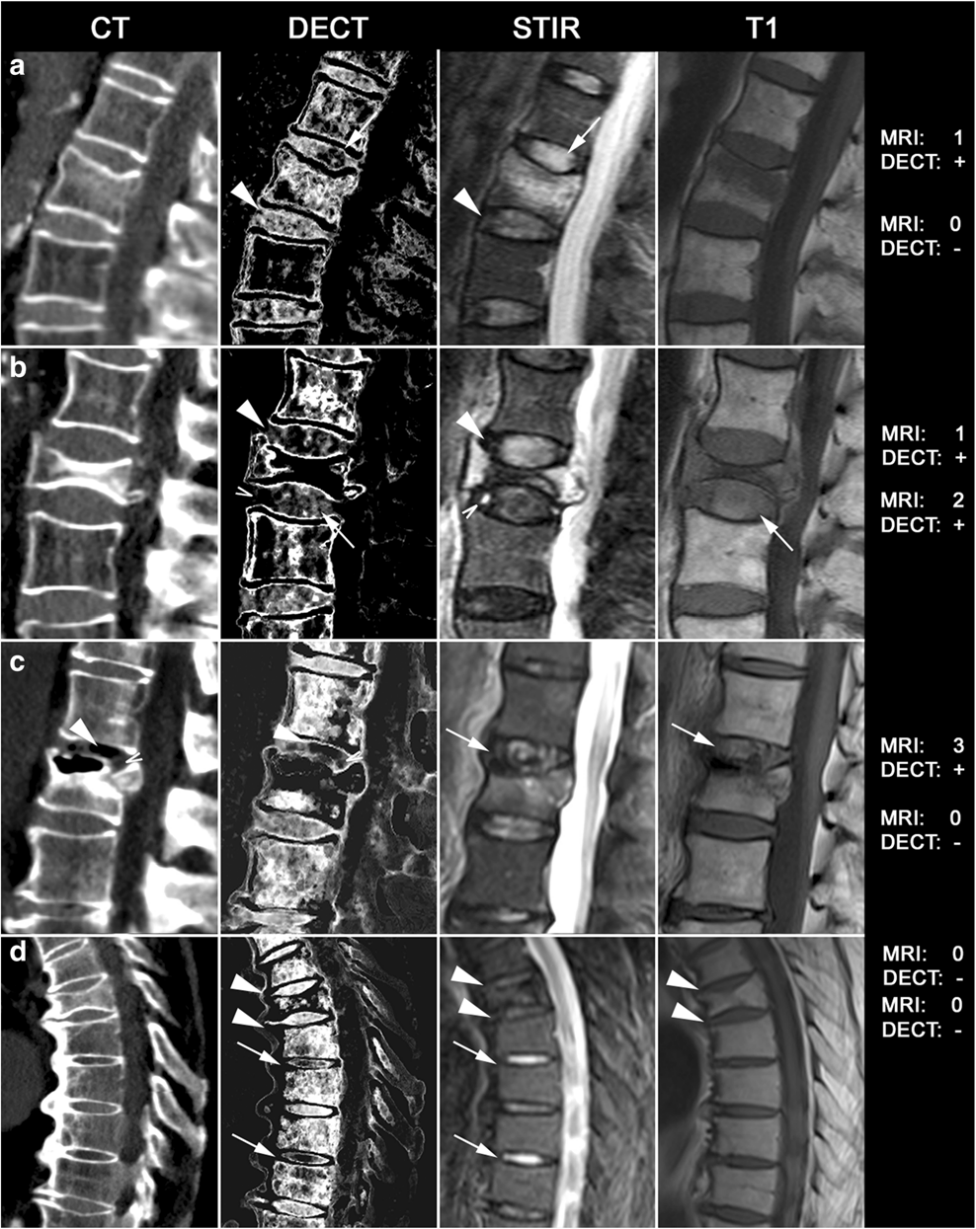
Imaging examples from various patients. Computed tomography (CT), dual-energy collagen maps (DECT), and magnetic resonance imaging short tau inversion recovery (MRI-STIR) and T1 images with combined scores across the three readers for MRI (0–3) and DECT (positive [+] or negative [−]). **a** A 79-year-old woman with a wedge fracture with bone marrow edema at L2. The cranial disk shows increased signal intensities in MRI-STIR with loss of the nuclear cleft in the posterior aspect (Sander grade 1) and a corresponding loss of collagen density in DECT, especially in the posterior part (arrow), whereas the caudal disk appears normal (arrowhead). **b** A 78-year-old man with a vertebral compression fracture with bone marrow edema at L2. Again, the cranial disk gives increased STIR signal intensity (grade 1) with a corresponding loss of collagen density in DECT (arrowhead). The caudal disk shows increased T1 signal intensity in the posterior aspect (and a corresponding loss of density in the DECT collagen map; arrow) and focal STIR hyperintensity in the anterior aspect, indicating rupture of fibrous annulus fibers accompanied by even greater signal loss in the DECT (open arrow). **c** A 66-year-old woman with an incomplete burst fracture at L3. The cranial disk shows high STIR signal, high T1 signal (arrow), and herniation through the endplate in CT (Sander grade 3). The DECT collagen map shows loss of density in areas with air (arrowhead) but also inside the disk material (open arrow). **d** A 68-year-old man with a wedge fracture at Th7. Adjacent disks show fairly normal signals in STIR and T1 without loss of collagen density in DECT (arrowheads). However, distant disks, not included in the analysis, show high STIR signal intensities (grade 1) and marked decreases in collagen density in DECT (arrows)

The results of the contingency table analysis comparing disk damage and endplate involvement in MRI and DECT are summarized in Tables 1 and 2. The resulting overall diagnostic accuracy of using DECT to depict disk injury was 0.81 compared with 0.75 for MRI when endplate involvement was the deciding factor. In DECT, SE varied with the grade of disk damage, ranging from 0.80 for grade 1 to 0.85 for grade 2 and 0.98 for grade 3. A collection of different images is shown in Fig. S1. Diagnostic accuracy varied between readers: SE was 0.89 for reader 1, 0.73 for reader 2, and 0.86 for reader 3, and SP was 0.93 for reader 1, 0.59 for reader 2, and 0.54 for reader 3, reflecting their experience levels. Fleiss’s *κ* was 0.41 (95% confidence interval, 0.34–0.47) for MRI and 0.51 (95% confidence interval, 0.44–0.57) for DECT. Imaging examples are shown in Fig. 2. A normal example of a DECT in a young patient (not part of the study collective) is shown in Supplementary Fig. S2. The analysis disk injury in the subgroup of patients under the age of 65 (17 patients; 72 disks) resulted in a SE of 0.89, SP of 0.9, PPV of 0.96, and NPV of 0.75.

**Table 1:** Contingency analysis for vertebral units with disk injury. Data are given with 95% confidence intervals. All values were calculated based on agreement between at least two of three readers using MRI as standard of reference

Vertebral unit disk injury	MRI+	MRI−	Total	SE	0.85	0.79 to 0.90
DECT+	165	25	190	SP	0.75	0.66 to 0.83
DECT−	29	76	105	PPV	0.87	0.82 to 0.91
Total	194	101	295	NPV	0.72	0.63 to .81

*SE* sensitivity, *SP* specificity, *PPV* positive predictive value, *NPV* negative predictive value.

**Table 2:** Contingency analysis for vertebral units with endplate involvement. Data are given with 95% confidence intervals. All values were calculated based on agreement between at least two of three readers using CT as standard of reference

Vertebral unit endplate involvement	CT+	CT−	Total	SE	0.70	0.62 to 0.76
MRI+	121	21	142	SP	0.83	0.75 to 0.89
MRI−	53	100	153	PPV	0.85	0.78 to 0.91
Total	174	121	295	NPV	0.65	0.57 to 0.73

*SE* sensitivity, *SP* specificity, *PPV* positive predictive value, *NPV* negative predictive value.

### Objective imaging parameters

**Fig. 3:**
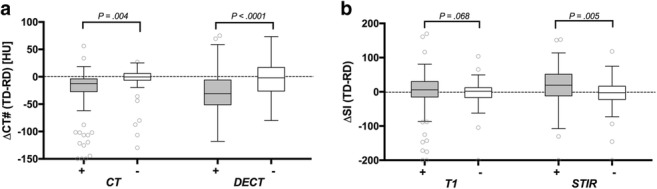
Differences between disks with (+, gray) and without (−, white) involvement on magnetic resonance imaging (MRI) compared with a normal-appearing reference disk. The CT number (CT#) or the respective signal intensity (SI) of the target disk (TD) minus the CT# or SI of the normal-appearing reference disk (RD) was calculated. **a** The CT and DECT collagen maps show decreases in HUs along with the damage. **b** The MRI-STIR shows increased signal intensity resulting from edema. Differences in T1 failed to reach significance

In CT imaging, attenuation in HUs measured lower for injured disks (detected by MRI; 69.1 ± 49.4 vs. 81.4 ± 41.7, *p* = 0.04). Similar results were obtained for the DECT collagen maps (80.3 ± 35.2 vs. 97.9 ± 41.0, *p <* 0.001). In MRI, STIR values were higher for damaged disks (148.1 ± 68.3 vs. 109.5 ± 53.8; *p* < 0.001) as they were for T1, but in the latter, significance was not reached (207.5 ± 90.7 vs. 186.8 ± 83.9, *p* = 0.07). Figure 3 shows results for the differences between target disks and reference disks.

## Discussion

To our knowledge, this is the first study analyzing the diagnostic accuracy of DECT for detecting disk abnormalities after vertebral fractures. Results show that DECT is suitable for detecting disk injuries in a cohort of patients with vertebral fractures. It has high SE and SP using MRI as the standard of reference, and SE increases with severity based on the Sander scale. Disk damage in DECT correlates better with endplate involvement in CT than disk damage in MRI. Surprisingly, DECT has higher interrater reliability compared to MRI when used to determine whether disk injury is present. Measurements in the ROI provide a satisfactory distinction between affected and unaffected disks in DECT, comparable to that achieved using STIR.

Overall, these results suggest that DECT can depict severe disk injuries with high diagnostic accuracy, thus providing important prognostic information [17, 18] in addition to its role in bone marrow edema assessment. Patients unable to undergo MRI for various reasons could benefit from this capability in the future. Information about disk injury is especially important in young patients with traumatic spinal fractures when deciding on a surgical approach and whether an additional anterior spinal fusion is necessary.

Conventional multidetector CT cannot provide information about disk injury [19]. However, DECT reconstructions are based on generic CT information. Therefore, our measurements using conventional CT images showed differences between affected and non-affected disks. By comparing low- and high-energy images, DECT can measure the effective atomic number in a voxel and thus improve characterization of the material. The densely packed collagen fibers in tendons and disks show specific properties in their interactions with X-rays. Hence, they can be detected and quantified using DECT [20]. We found that the collagen concentration in vertebral disks decreases with injury.

In this study, DECT showed superior interrater agreement and better correlation with endplate involvement, the most commonly suspected cause of disk injury [8, 21–23]. The uncertainty of our standard of reference might explain why we found a rather low SP. The lower interrater reliability might indeed be attributable to the reader taking more information into account when using MRI (T1 and STIR signal intensity and morphology) compared to DECT (collagen map and morphology) for evaluation. This might be especially difficult for less experienced readers. Furthermore, the Sander scale (especially grades 1 and 2) rating remains controversial in our rather aged collective of patients. Persistent nucleus pulposus might imitate an increased T2 signal compared to disks affected by age-related decreases in water content. Therefore, our standard of reference might be prone to false-positive detections, thus resulting in decreased SP when using DECT. Furthermore, there are other causes of discal T1 increased signal changes than bleeding. However, during scoring, we took great care in evaluating the cleft sign and the Sander scale, as validated in the literature. Moreover, our limited experience with DECT in young patients shows that this technique might be unaffected by these changes (see Fig. S1).

Nonetheless, our results cannot be readily transferred to patients younger than those in our cohort. Fluid inside the nucleus pulposus decreases with age [24, 25], and collagen content is lower in lumbar compared to that in thoracic disks [26]. In fact, the readers noted that in the lumbar region, the annulus fibrosus was highly important, whereas in the thoracic region, each loss of HUs in the collagen map could be considered positive without loss of SP. However, in our subgroup analysis of patients younger than 65 years, the diagnostic accuracy increased compared to the whole collective, indicating that this technique might be influenced by age-related disk changes. Furthermore, these results cannot be easily transferred when using software from different vendors without possibly changing the dual-energy gradient in collagen or taking a different approach to DE imaging (e.g., with a tin filter for a different energy separation). However, this question should be targeted in future studies.

Although this is the first study of DECT collagen imaging of the spine, other studies have shown its capabilities in other anatomic regions. Peltola et al reported high SE (79%) and SP (100%) when using it to detect anterior cruciate ligament tears [27]. Mallinson et al diagnosed Achilles tendinopathy with a technique similar to the one we used [12]. Kumar et al detected and quantified myocardial fibrosis [20]. However, overall, published data on this technique remain limited.

Recent studies demonstrate that vertebral fractures are largely underdiagnosed by radiologists [28, 29], especially when the image presents with only a mild degree of wedging or biconcavity because normal variations, developmental abnormalities, degenerative changes, and other conditions have to be considered. Therefore, a qualitative approach for identifying fractures offers low reproducibility, particularly when performed by inexperienced observers. However, the semiquantitative method based on a visual assessment of vertebral morphology and of vertebral height reduction provides reasonable reproducibility, SE, and SP when performed by an expert radiologist [15]. Therefore, we used this technique to assess the creation of entry criteria for disks at risk of injury (target disks).

The limitations of this study must be discussed. We included only patients aged at least 50 years with vertebral fractures. Therefore, these results cannot be transferred to posttraumatic fractures or a younger patient population. Histology was not available for all patients because only a minority underwent surgical treatment. However, MRI is an accepted modality for evaluating disk abnormalities [30]. Our DECT scanning protocol was optimized for the depiction of bony structures in terms of radiation exposure. This might have influenced image quality of the collagen maps. Better results might be achieved with greater radiation exposure. Furthermore, DECT cannot separate various degrees of disk damage; thus, the ultimate impact on the treatment decision remains unclear. Additionally, DECT does not directly identify edema or bleeding within a disk but rather a loss of collagen matrix per volume unit which can be caused by a decrease in HUs due to edema or a change of the dual-energy gradient by adding elements with higher effective atomic numbers such as iron in blood. However, there could be other causes of intradiscal collagen changes after trauma that could be detected by DECT but not by MRI. This cannot be determined without histologic analysis. Furthermore, the impact of disk degeneration on the DECT images was not assessed.

We showed that DECT with reconstruction of collagen maps using a three-material decomposition algorithm can detect vertebral disk involvement in elderly patients with vertebral fractures. It provides reasonably high SE and SP and a better correlation with endplate involvement, and it also provides an interrater reliability that is not inferior to that of MRI. Future studies should aim to use a histologic reference standard for a better interpretation of findings.

## Electronic supplementary material

ESM 1 (DOCX 4425 kb)

## Abbreviations

DECT: Dual-energy computed tomography
ROI: Region of interest
SD: Standard deviation
STIR: Short tau inversion recovery
