# Supplementary material for: Disk injury in patients with vertebral fractures—a prospective diagnostic accuracy study using dual-energy computed tomography
Source: Eur Radiol. 2019 Jan 16;29(8):4495–502. doi: 10.1007/s00330-018-5963-4 (PMC6610270; doi:10.1007/s00330-018-5963-4)
Supplement: Supplementary file 1 — (DOCX 4425 kb) [file 330_2018_5963_MOESM1_ESM.docx]

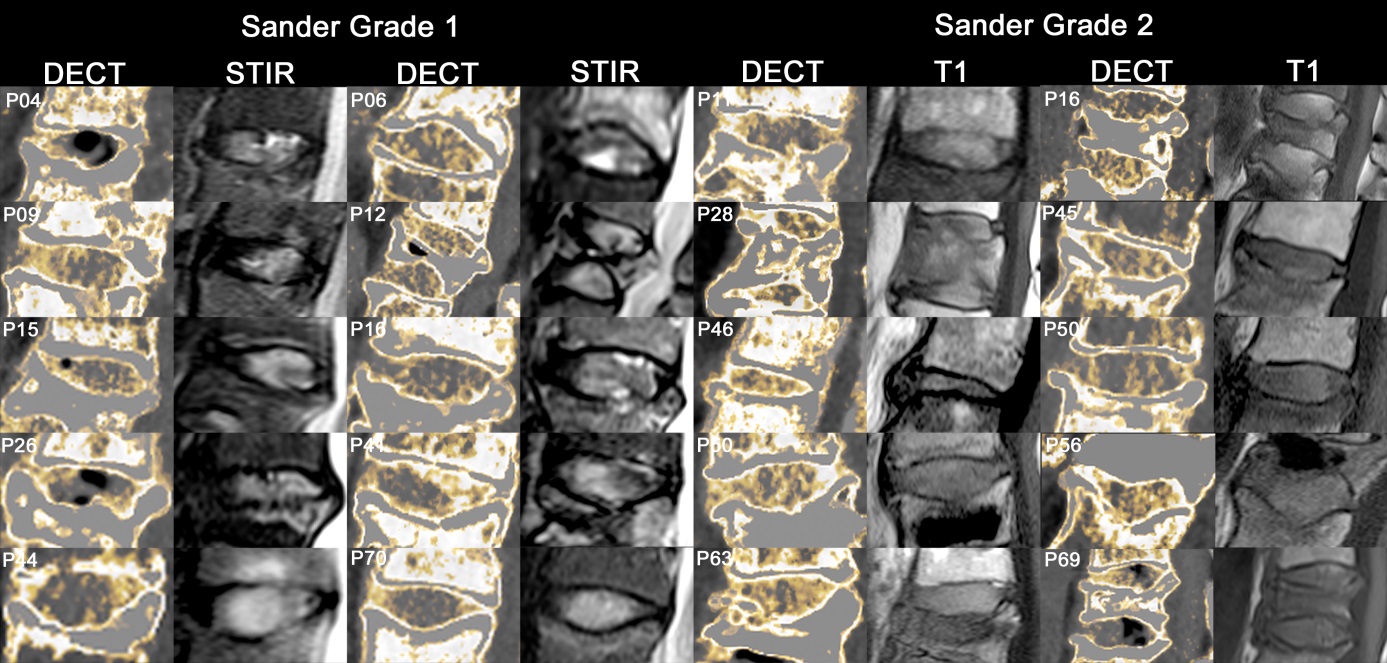


Fig. S1 – Collection of positive disks. Dual-energy computed tomography (DECT) collagen maps of disks with high T2-signal in magnetic resonance imaging (MRI) short-tau inversion recovery (STIR) sequence (grade 1 on the Sander scale) and high T1-signal in MRI (grade 2 on the Sander scale).


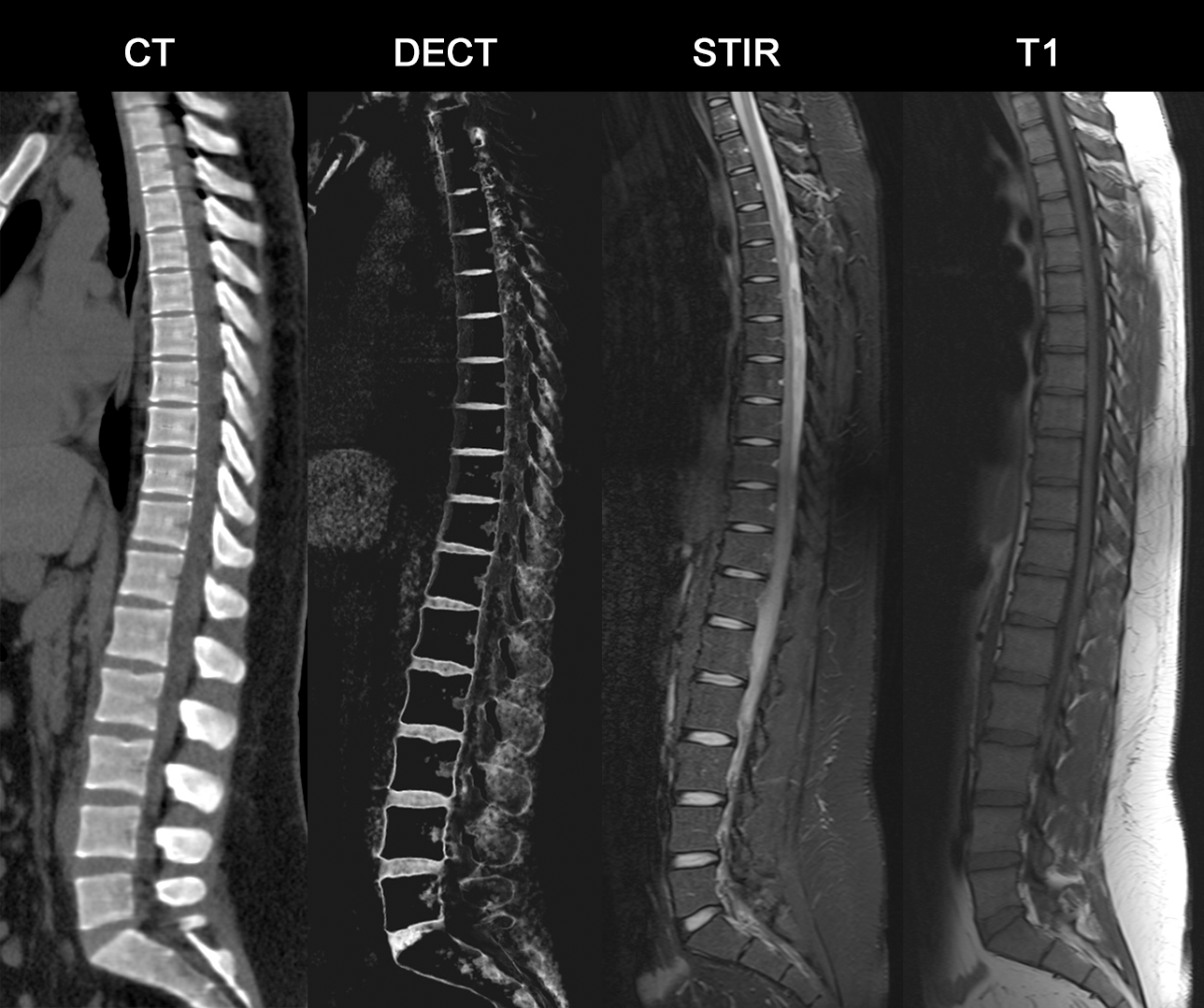


Fig. S2 – Example of a young patient. A 21-year-old patient undergoing total spine magnetic resonance imaging (MRI) and low-dose dual-energy computed tomography (DECT) for a different study. This patient also gave written informed consent. The DECT collagen maps were reconstructed using an algorithm and software-settings identical to those used in the study. They show high collagen content in all thoracic and lumbar disks despite persistent nucleus pulposus and normal signal intensities in MRI. However, this has not been proven in a larger patient cohort.
